# Multimodal prehabilitation in patients with non-small cell lung cancer undergoing anatomical resection: protocol of a non-randomised feasibility study

**DOI:** 10.1186/s13741-023-00326-y

**Published:** 2023-07-19

**Authors:** Charlotte Johanna Laura Molenaar, Erik Martin Von Meyenfeldt, Carlijn Tini Ireen de Betue, Rosaline van den Berg, David Wouter Gerard ten Cate, Goof Schep, Magdolen Youssef-El Soud, Eric van Thiel, Nicky Rademakers, Sanne Charlotte Hoornweg, Gerrit Dirk Slooter, Frank van den Broek, Geertruid Marie Heleen Marres, Dutch Prehab Lung Research Group, Loes van de Voort, Frank de Kort, Chris de Jongh, Cathrin van Erven, Mirjam Staffeleu–Noodelijk, Els Driessen, Marieke van de Wal, Netty de Graaff, Anouk van Limpt, Maaike Scholten-Bakker

**Affiliations:** 1 Department of Surgery, Máxima MC, Veldhoven, the Netherlands; 2 Department of Surgery, Albert Schweitzer Hospital, Dordrecht, the Netherlands; 3 Science Office, Albert Schweitzer Hospital, Dordrecht, the Netherlands; 4 Department of Sports Medicine, Máxima MC, Veldhoven, the Netherlands; 5 Department of Pulmonology, Máxima MC, Veldhoven, the Netherlands; 6 Department of Pulmonology, Albert Schweitzer Hospital, Dordrecht, the Netherlands; 7 Physiotherapy Department, Máxima MC, Veldhoven, the Netherlands; 8 Physiotherapy Department, Albert Schweitzer Hospital, Dordrecht, the Netherlands

**Keywords:** Prehabilitation, Preoperative intervention, Enhanced recovery after thoracic surgery, Non-small cell lung cancer, Anatomical resection, Lung surgery, Functional capacity, Physical conditioning, Postoperative outcome, Feasibility

## Abstract

**Background:**

The preoperative period can be used to enhance a patient’s functional capacity with multimodal prehabilitation and consequently improve and fasten postoperative recovery. Especially, non-small cell lung cancer (NSCLC) surgical patients may benefit from this intervention, since the affected and resected organ is an essential part of the cardiorespiratory fitness. Drafting a prehabilitation programme is challenging, since many disciplines are involved, and time between diagnosis of NSCLC and surgery is limited. We designed a multimodal prehabilitation programme prior to NSCLC surgery and aimed to conduct a study to assess feasibility and indicative evidence of efficacy of this programme. Publication of this protocol may help other healthcare facilities to implement such a programme.

**Methods:**

The multimodal prehabilitation programme consists of an exercise programme, nutritional support, psychological support, smoking cessation, patient empowerment and respiratory optimisation. In two Dutch teaching hospitals, 40 adult patients with proven or suspected NSCLC will be included. In a non-randomised fashion, 20 patients follow the multimodal prehabilitation programme, and 20 will be assessed in the control group, according to patient preference. Assessments will take place at four time points: baseline, the week before surgery, 6 weeks postoperatively and 3 months postoperatively. Feasibility and indicative evidence of efficacy of the prehabilitation programme will be assessed as primary outcomes.

**Discussion:**

Since the time between diagnosis of NSCLC and surgery is limited, it is a challenge to implement a prehabilitation programme. This study will assess whether this is feasible, and evidence of efficacy can be found. The non-randomised fashion of the study might result in a selection and confounding bias. However, the control group may help putting the results of the prehabilitation group in perspective. By publishing this protocol, we aim to facilitate others to evaluate and implement a multimodal prehabilitation programme for surgical NSCLC patients.

**Trial registration:**

The current study is registered as NL8080 in the Netherlands Trial Register on the 10th of October 2019, https://www.trialregister.nl/trial/8080. Secondary identifiers: CCMO (Central Committee on Research Involving Human Subjects) number NL70578.015.19, reference number of the Medical Ethical Review Committee of Máxima MC W19.045.

## Background

Worldwide, lung cancer is the leading cause of cancer death and has the highest incidence amongst cancer types (Bray et al. 2018). The preferred curative treatment option for resectable non-small cell lung cancer (NSCLC) is anatomical resection combined with mediastinal lymph node dissection. However, surgery and postoperative complications contribute to significant morbidity. Major pulmonary complications negatively impact long-term outcomes, including a reduced disease-free survival (Wang et al. 2017). Furthermore, pulmonary complications affect patient-centred outcomes and healthcare costs (Templeton and Greenhalgh 2019).

In the past decades, the preoperative period has been used more and more to prepare patients for surgery by means of prehabilitation. These multimodal prehabilitation programmes aim to enhance functional capacity, prepare patients for treatment and consequently and improve outcome. The first official guidelines for enhanced recovery after lung surgery by the Enhanced Recovery After Surgery (ERAS®) Society and the European Society of Thoracic Surgeons (ESTS) strongly recommend prehabilitation for patients with borderline lung function or exercise capacity (Batchelor et al. 2019). Several systematic reviews and meta-analyses on the effect of various preoperative interventions prior to lung cancer surgery have recently been published, reporting a reduction in postoperative (pulmonary) complications and length of hospital stay (Cavalheri and Granger 2017; Kendall et al. 2018; Rosero et al. 2019; Steffens et al. 2018). In addition to these benefits, patients deemed unfit for surgery, based on their pulmonary function test and exercise test results, could potentially become a surgical candidate with prehabilitation (Mahendran and Naidu 2018).

To date, delaying surgery for patients with lung cancer to facilitate prehabilitation cannot be recommended, because cancer could potentially progress (Cavalheri and Granger 2017; Mahendran and Naidu 2018). The Dutch national guideline for the treatment of NSCLC states that 80% of patients should be operated within 2 weeks after completion of the diagnostic phase (National guideline non-small cell lung cancer n.d.). This is monitored by the Dutch Institute for Clinical Auditing (DICA) through the compulsory Dutch Lung Cancer Audit — Surgery (DLCAs), which records and benchmarks the percentage of operations performed within 3 weeks from the time of the multidisciplinary team meeting confirming diagnosis (Dutch Institute and for Clinical Auditing n.d.). The window of opportunity prior to surgery to optimise patients is therefore limited, yet meaningful improvements from such interventions have been demonstrated in a recently published randomised controlled trial with a multimodal prehabilitation programme in a home-based setting with a 2-week window before surgery (Liu et al. 2020).

The aim of this multicentre, non-randomised study is to evaluate feasibility and assess indicative evidence of efficacy of a supervised high-intensity multimodal prehabilitation programme for surgical NSCLC patients in the Dutch healthcare system. By publishing the protocol of our study, we aim to encourage colleagues to implement a similar programme in their own facilities more easily and possibly adjust it to use for other patient populations as well. Publication of this protocol enables us to describe the programme more detailed, than when reported simultaneously with the final results of our study. Besides, this publication will contribute to transparency in the conduction of studies.

## Methods

### Study design and setting

Our study is a non-randomised feasibility study, approved by the Medical Ethical Review Committee of Máxima MC (MMC) (reference number: W19.045) and the Institutional Review Board of the Albert Schweitzer Hospital (ASz). Patient recruitment commenced in January 2020 and was temporarily interrupted between March 2020 and June 2020 due to the coronavirus disease 2019 (COVID-19) pandemic. The two Dutch teaching hospitals have expertise regarding prehabilitation programmes for oncological surgery and enhanced recovery after thoracic surgery.

For this protocol, the reporting standards of the SPIRIT (Standard Protocol Items: Recommendations for Interventional Trials) checklist, adapted for pilot and feasibility studies, were used as guidance (Thabane and Lancaster 2019).

### Participants

All consecutive patients meeting the inclusion criteria will be asked to participate in the multimodal prehabilitation programme. Patients are deemed eligible when ≥ 18 years of age, with pathologically confirmed or suspected NSCLC and an indication for anatomical lung resection. Furthermore, a straightforward preoperative work-up is needed to ensure patients can enter the programme directly after diagnosis in order to use the window of opportunity between diagnosis and surgery as efficient as possible. Also, the ability to provide written informed consent is needed for inclusion. Eligible patients not willing to participate in the multimodal prehabilitation programme will be asked to participate in the control group. The control group may provide insight in the course of functional capacity perioperatively and will enable comparison with the prehabilitation group.

Exclusion criteria comprise a contra-indication for training, renal insufficiency (defined as estimated glomerular filtration rate (eGFR) < 60 ml/min/1.73 m^2^) or participation in other trials that may interfere. Referred patients from another hospital with the diagnostic phase already completed will be excluded, for logistical reasons, travel distance and the probability that these factors may affect feasibility of the programme. If a contra-indication for training will become apparent after signing informed consent and following baseline assessment, we intend to exclude the subject from participation for safety reasons. Furthermore, if the postoperative pathology results will not confirm the diagnosis NSCLC, the data of this patient will be included in the analysis; however, we intend to recruit an extra patient to finally include a total of 40 patients with confirmed NSCLC, per centre ten patients in the prehabilitation group and ten in the control group. All assessed patients will be logged.

**Fig. 1:**
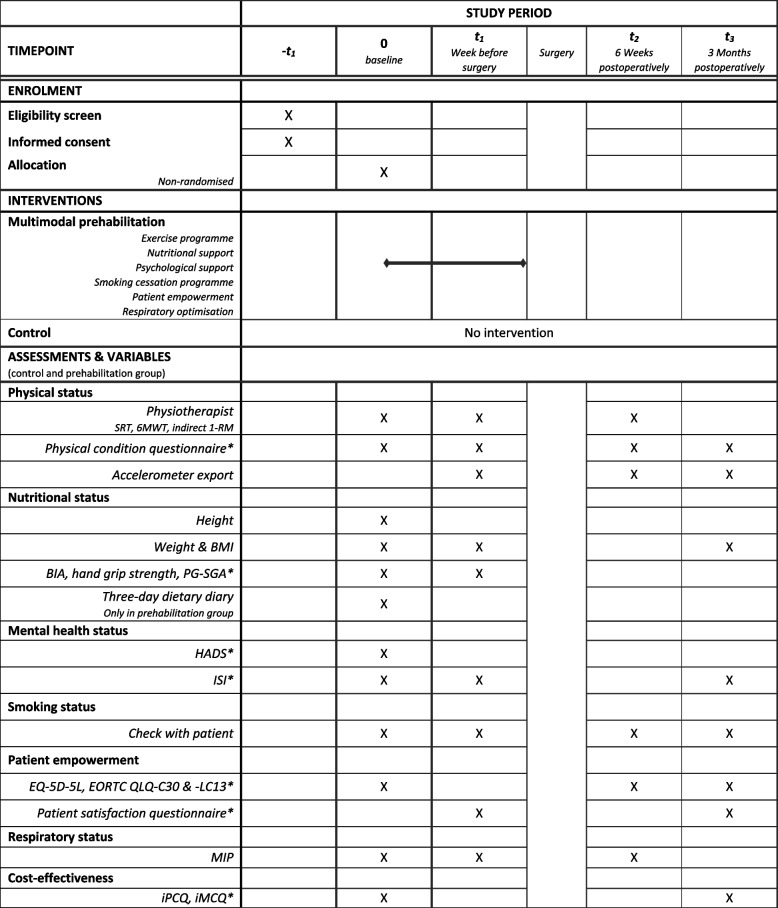
SPIRIT-derived overview of time points, assessments and recorded variables. 6MWT, 6-min walk test; BIA, bio-impedance electrical analysis; BMI, body mass index; EORTC QLQ-LC13, European Organisation for Research and Treatment of Cancer, quality of life of cancer patients — lung cancer-specific module; EORTC QLQ-C30, European Organisation for Research and Treatment of Cancer, quality of life of cancer patients module; EQ-5D-5L, general health-related quality of life questionnaire; HADS, Hospital Anxiety and Depression Scale; iMCQ, Institute for Medical Technology Assessment (iMTA) Medical Consumption Questionnaire; Indirect 1-RM strength test, indirect 1-repetition maximum; iPCQ, iMTA Productivity Cost Questionnaire; ISI, Insomnia Severity Index; MIP, maximal inspiratory pressure; PG-SGA, Patient-Generated Subjective Global Assessment; SRT, steep ramp test. *All questionnaires will be sent by e-mail

The sample size of 40 participants for this feasibility study is based on a previously performed pilot study in colorectal cancer patients (Rooijen et al. 2019). This study was able to assess feasibility and indicative evidence of efficacy with a similar sample size. Patients will be selected and informed about the study during a visit to their treating pulmonologist in the outpatient clinic. Informed consent will be obtained by a member of the research team during a face-to-face consult in the outpatient clinic prior to baseline assessment. We intend to conduct the study and finish follow-up between January 2020 and January 2022. The SPIRIT-derived Fig. 1 displays an overview of the time points, assessments and recorded variables.

### Assessments

Both groups will be assessed at baseline, the week before surgery, 6 weeks postoperatively and 3 months postoperatively.

#### Physical status

Patients will undergo an assessment by a physiotherapist to determine functional capacity and will be requested to fill in a questionnaire estimating physical condition.

A questionnaire to estimate physical condition (FitMáx^©^) will be used to estimate the maximum oxygen uptake expressed as VO2max. This questionnaire is developed by one of the authors with an expertise in exercise physiology (G. Schep) and is validated in 700 subjects varying from healthy status to those with pulmonary disease, heart disease or cancer (Meijer et al. 2022). Results pointed out that the questionnaire had a correlation of 0.93 with VO2max determined with the Cardiopulmonary Exercise Test (CPET), the gold standard to determine functional capacity. The questionnaire will be sent to patients by email at baseline, prior to surgery and 6 weeks as well as 3 months postoperatively.

Additionally, the physiotherapist will determine functional capacity using the 6-min walk test (6MWT), steep ramp test (SRT) and indirect one-repetition maximum (1-RM) strength test. Each test will be followed by a break of approximately 10 min. The tests will be completed at baseline, the week before surgery and 6 weeks postoperatively. The walking distance in metres during the 6MWT is determined by counting the number of times a patient walks back and forth on a straight 30-m track during 6 min. Patients will be encouraged and will receive feedback according to a standardised schedule (according to the American Thoracic Society). Patients will be allowed to use any walking device, if necessary (ATS Committee on Proficiency Standards for Clinical Pulmonary Function Laboratories 2002). With the SRT, functional capacity is determined as maximum short exercise capacity (MSEC) on a calibrated bicycle ergometer (Backer et al. 2007; Stuiver et al. 2017). The test starts with 3 min of warming up at 0 W, followed by an increase of resistance by 25 W per 10 s while maintaining 70–80 rpm. The incremental phase continues until exhaustion occurs, defined as < 60 rpm (Meyer et al. 1997). The MSEC is expressed as total Watt achieved until exhaustion. To determine strength, the 1-RM strength test is the golden standard. In this study, the indirect 1-RM strength test, defined as the weight a person can lift at least two and maximum ten times, will be used because the 1-RM can be strenuous and may cause injuries. The 1-RM is calculated using the Brzycki equation: 1-RM = (used weight/(1.0278–0.0278*number of repetitions) (Reynolds et al. 2006).

To monitor the compliance to the out-of-hospital exercises (described in the “interventions” section) in the intervention group and monitor physical activity in the control group, a hip-worn activity monitor (PAM AM 300, Pam Private Company, Oosterbeek, the Netherlands) will be provided to participants in both groups for the duration of the study. Patients will be asked to wear the activity monitor from the baseline assessment until 3 months after surgery, during daytime from getting out of bed until going to bed.

#### Nutritional status

Prior to the initial consultation by the dietitian, only the prehabilitation group will complete a 3-day dietary diary to estimate energy and protein intake.

To assess nutritional status, the following measurements will be performed at baseline and the week prior to surgery in both groups. Weight and height will be measured to calculate the body mass index (BMI). To screen for malnutrition, the Patient-Generated Subjective Global Assessment (PG-SGA) will be completed at baseline and the week prior to surgery (Ottery 1996; Sealy et al. 2018). To determine body composition, a standardised bioelectrical impedance analysis (BIA) with a single-frequency 50-kHz impedance metre (Bodystat 500, Bodystat, Isle of Man, UK) will be performed. Different equations will be used to calculate fat-free mass (FFM): Kyle when BMI < 30 (Kyle et al. 2003), Horie with a BMI ≥ 30 (Horie et al. 2011) and Rutten in case of chronic obstructive pulmonary disease (Rutten et al. 2010). Finally, the hand grip strength will be determined by the dietitian with a hand-held hydraulic dynamometer (Jamar, Patterson Medical, Warrenville, IL, USA). The measurement will be repeated three times, and the highest result of both hands will be recorded.

#### Mental health status

The psychological assessment be determined using the Hospital Anxiety and Depression Scale (HADS) (Zigmond and Snaith 1983), consisting of a 7-item subscale to assess complaints of anxiety and depression, and the Insomnia Severity Index (ISI) (Bastien et al. 2001), assessing sleeping disturbances. In the prehabilitation group only, during a consultation with a clinical psychologist, the results of these questionnaires will be discussed. Since the score of the HADS is not expected to change in a limited period of 2 to 3 weeks, it will only be completed at baseline. The ISI will be completed at baseline, the week prior to surgery and 3 months after surgery.

#### Quality of life: assessing patient empowerment

Quality of life will be assessed at baseline, 6 weeks and 3 months after surgery, using the following questionnaires sent by email:

- EQ-5D-5L (EuroQol group, 5-level general health-related quality-of-life questionnaire (Herdman et al. 2011; Euroqol Group n.d.)
- EORTC QLQ-C30 (European Organisation for Research and Treatment of Cancer; quality of life of cancer patients (Aaronson et al. 1993; European Organisation for Research and Treatment of Cancer n.d.)
- EORTC QLQ-LC13 (European Organisation for Research and Treatment of Cancer; quality of life of cancer patients — lung cancer specific (European Organisation for Research and Treatment of Cancer n.d.; Bergman et al. 1994)

Additionally, a patient satisfaction questionnaire assessing all items of the programme is drafted by the research team and will be sent by email to both groups the week prior to surgery and 3 months postoperatively.

#### Respiratory status

Pulmonary functioning will be assessed according to standard of care. Additionally, the maximal inspiratory pressure (MIP) will be measured with the MicroRPM (respiratory pressure meter, CareFusion, San Diego, CA, USA) in order to determine the intensity of the inspiratory muscle training, which will be elaborated further in the “interventions” section. The MIP will be measured three times, aiming for a difference less than 5%. The highest pressure will be recorded and used for the programme. The MIP will be determined at baseline, the week before surgery and 6 weeks postoperatively.

#### Cost-effectiveness

Finally, to estimate the cost-effectiveness of the multimodal prehabilitation programme, a shortened institute for Medical Technology Assessment (iMTA) questionnaire (Bousema et al. 2018) will be used, consisting of the iMTA Productivity Cost Questionnaire (iPCQ) and the iMTA Medical Consumption Questionnaire (iMCQ). Patients will be requested to fill out the iPCQ and iMCQ at baseline and 3 months after surgery.

### Interventions

The 3-week multimodal prehabilitation programme commences after the baseline assessment and finishes 3–4 days before surgery. The programme contains six pillars: exercise programme, nutritional support, psychological support, smoking cessation, patient empowerment and respiratory optimisation (disease specific optimisation).

#### Exercise programme

The exercise programme consists of in-hospital, supervised training on three nonconsecutive days per week, for about 60 min, consisting of high-intensity interval training (HIIT) endurance training and strength exercises. Patients will be supervised by a physiotherapist, specialised in oncology and preferably train in peer groups if permitted by the periodicity of inclusion of subjects and existing COVID-19 measures. The intensity of the exercise programme will be individualised based on the baseline assessment and will be continuously evaluated by the physiotherapist during the programme.

The HIIT will be performed on a bicycle ergometer and consists of two series of eight repetitions of uninterrupted cycling. One repetition consists of 30 s of strain, set on 65% of the MSEC measured with the SRT, followed by 30 s of rest being 30% of the MSEC. Intensity of the HIIT will be monitored and adjusted, if necessary: if a patient scores ≤ 11 on the Borg scale (Achttien et al. 2011), the intensity will be increased by 5%; intensity will be decreased by 5% when the Borg score is ≥ 15 or when a patient will not be able to complete the eight repetitions.

The supervised strength exercises will be performed on fitness devices, and the intensity will be based on the 1-RM strength test calculation at baseline. The intensity will start with 65% of 1-RM in week 1, will increase to 70% in week 2 and if time allows 75% in week 3. Strength training will consist of two series of ten repetitions of the following exercises, targeting major muscle groups: lateral pull down, leg press, chest press and low row.

Finally, patients will be instructed by the physiotherapist to perform out-of-hospital exercises — like walking or cycling, according to patient preference — with low to moderate intensity, on the remaining 4 days of the week for at least 60 min per day.

#### Nutritional support

Nutritional status will be optimised through nutritional counselling by a registered dietitian. Based on the baseline assessment, the dietitian will provide a tailored dietary advice, including energy and protein requirements. Energy intake will be based on the calculated energy expenditure. The recommended daily protein intake is 1.9–2.2 g of protein/kilogram FFM (as determined with BIA). When FFM cannot be calculated, actual body weight will be used with a daily protein recommendation of 1.5–1.8/kg.

All patients in the prehabilitation group will receive a high-quality protein powder supplement containing whey and casein proteins, providing 10 g of essential amino acids — of which 2 to 3 g of leucine — per portion of 30 g (Refit®-TMP-90-Shake, Friesland Campina Domo, Amersfoort, the Netherlands). Patients will be instructed to ingest 30 g of supplement within 1 h after the in-hospital supervised training and daily 1 h before going to sleep.

Additionally, vitamin D and multivitamin supplements will be provided for daily use. Vitamin D dosage depends on age, gender, skin type and sun exposure and is based on the guideline of the Health Council of the Netherlands (Vitamin D guideline, Health Council of the Netherlands n.d.).

#### Psychological support

Psychological support will consist of a 45-min explorative consultation by a clinical psychologist. During this consult, burden of the disease and accompanied treatment as well as the patient’s coping strategies will be assessed, and when indicated, empowerment or psychoeducation will be provided. The clinical psychologist will determine whether follow-up sessions are needed during the perioperative phase and/or referral to a psychiatrist is indicated and will act accordingly. Furthermore, breathing and relaxation techniques, assisted with audio, will be provided to the patients.

#### Smoking status

If applicable, patients will be offered to follow a smoking cessation programme. This consists of counselling and nicotine replacement therapy and is outsourced to a specialised institute in the Netherlands (SineFuma Private Company, Breda, the Netherlands) (SineFuma n.d.).

#### Patient empowerment

To maximise patient empowerment, participants will be optimally informed and educated about the prehabilitation programme, the surgical care pathway and their own contribution. An information booklet containing information on all interventions of the programme will be handed out; a logbook will be provided to register all study-related activities.

#### Respiratory optimisation (disease-specific optimisation)

Disease-specific optimisation is directed at the affected organ system: the respiratory system in the case of NSCLC. During the in-hospital supervised sessions, patients will perform inspiratory muscle training (IMT). An IMT device (Philips-Respironics Threshold IMT, Philips, Eindhoven, the Netherlands) will be provided to the patient and intensity will be based on the MIP assessment at baseline. The training starts at 40% of the MIP and will increase to at least 50%. The patient will receive instructions from the physiotherapist how to perform the exercise at home. Training will be performed twice daily for 15 min. Additionally, breathing and sputum clearance techniques will be taught by the physiotherapist prior to surgery.

### Outcome measures

#### Primary outcomes

The main primary outcome of this study is feasibility of the prehabilitation programme. Feasibility is defined as ≥ 80% protocol adherence (per intervention) of at least 80% of the participants in the prehabilitation group. To determine feasibility, we will collect the following data: number of eligible patients, number of patients enrolled, number of drop outs, number of attended supervised training sessions (per patient), patient’s logging booklet (number of out-of-hospital low to moderate training, recorded intake of nutritional supplements) and activity monitor data. The second primary outcome is functional capacity. Functional capacity will be determined at baseline, the week prior to surgery and 6 weeks postoperatively. Change over time compared to baseline within and between groups will be determined.

#### Secondary outcomes

The following secondary outcomes will be studied:

- Programme evaluation as a whole and per intervention (based on compliance, patient satisfaction and experience of the research team)
- Effect on clinical outcome: 30-day mortality, length of hospital stay, 30-day complication rate expressed as the comprehensive complication index (Slankamenac et al. 2013) and readmission rate. This will be compared to the control group and a historical Dutch cohort as recorded in the DLCAs (Dutch Institute and for Clinical Auditing n.d.).
- Cost-effectiveness of the prehabilitation programme
- Efficacy of the programme on nutritional and mental health status, smoking cessation and quality of life compared to baseline and control group
- Correlation between VO2max estimated with the physical condition questionnaire (FitMax^©^) and as determined with the SRT and, if applicable, CPET

### Data handling

With the use of an electronic case report form (eCRF), a database was built in castor electronic data capture (EDC) (Castor EDC n.d.). Castor EDC complies with Title 21 of the Code of Federal Regulations Part 11, General Data Protection Regulation (GDPR) and is fully ISO (International Organization for Standardization) 27001, 27002 and 9001 certified. When a new patient will be registered in castor EDC, patient codes are automatically generated, complying with the GDPR. Most of the data from the assessments will be entered directly into the eCRF. The remaining data will be entered by members of the research team in both centres. To guarantee the quality of this study, monitoring will be executed by an independent party appointed by the sponsor. All data saved in Castor EDC and source files will be stored for a period of 15 years.

### Statistical analysis

Because of the sample size of the group, descriptive data will be presented as median (interquartile range and/or range). Categorical data will be presented as numbers and percentages. Results of validated questionnaires will be calculated, and missing data will be handled using the instructions from the original authors. Missing data which will be used in the definition of feasibility will be considered as “not performed”.

Feasibility will be descriptively evaluated (group level and patient level), both dichotomously (yes/no) and as percentage of the total number of offered interventions.

Comparative outcomes, such as functional capacity, between the different test moments will be compared within the group participating in the multimodal prehabilitation programme and within the control group with the Friedman test and between groups with the Mann–Whitney *U*-test. Nonparametric tests will be used because of the small sample size. Dichotomous data will be compared between groups with chi-square test. Mixed model analyses will be used to evaluate functional capacity over time.

## Discussion

Since many disciplines are involved and time is limited, multimodal prehabilitation is a logistic challenge. It is widely studied in patients with colorectal cancer and deemed feasible and effective (Rooijen et al. 2019; Trepanier et al. 2019; Scheede-Bergdahl et al. 2019). However, in the Netherlands, the recommended treatment interval for NSCLC is shorter compared to colorectal cancer due to tumour biology and mutual agreements amongst scientific societies and the DICA.

However, especially, the NSCLC population may particularly benefit from prehabilitation. The pulmonary system is one of the determinants of cardiorespiratory fitness, a measure interpreted as the reflection of total body health (Ross et al. 2016), as well as the organ system affected by NSCLC. Furthermore, the lung is the organ that is resected during surgery, thereby further compromising cardiorespiratory fitness in itself. Although improving cardiorespiratory fitness preoperatively will not prevent a decrease of functional capacity postoperatively, prehabilitation may limit the extent.

This protocol is a result of an extensive multidisciplinary project. Two clinical groups with expertise in perioperative recovery optimisation developed a protocol based on experience and available evidence, combining various interventions within a short timeframe. In order to make a patient-friendly programme, all disciplines need to optimise and harmonise planning.

To our knowledge, only one randomised controlled trial with a multimodal prehabilitation programme in a NSCLC population has been published; however, this concerned a non-supervised home-based programme with less interventions compared to our study (Liu et al. 2020). In the Netherlands, we experience a growing interest in prehabilitation prior to surgery, yet to date, there are no formal public prehabilitation programmes available for NSCLC surgery patients.

The results of prehabilitation are promising thus far in other cancer types (Minnella et al. 2021; Ploussard et al. 2020; Swaminathan et al. 2020). Since one of the primary aims of our study is to determine the feasibility — the main perceived barrier for application — of our multimodal prehabilitation programme, a study design based on randomisation was deemed suboptimal. Although a similar pilot study in patients with colorectal cancer did not find a better baseline functional capacity in the prehabilitation group compared to the control group (Rooijen et al. 2019), a selection and confounding bias may occur in our study. The control group in our study will put the prehabilitation group in perspective, since this enables us to determine the “normal” course in the perioperative period using data from the assessments and accelerometers. Additionally, studying the control group may help assess feasibility further.

In conclusion, with this study, we aim to examine if an extensive, mainly in-hospital multimodal prehabilitation programme is feasible in the limited preoperative period of maximum 3 weeks, as determined by our national guideline. Furthermore, we aim to study whether patient optimisation can be achieved. By publishing this protocol, we provide a detailed description of the programme to improve transparency. Hopefully, this may help other hospitals to shorten the process of multidisciplinary set-up and implement a multimodal prehabilitation programme more easily.

## Data Availability

Not applicable.

## Abbreviations

(Indirect) 1-RM: (Indirect) one-repetition maximum
6MWT: 6-Min walk test
ASz: Albert Schweitzer hospital
BIA: Bioelectrical impedance analysis
BMI: Body mass index
BSc: Bachelor of Science
Castor EDC: Castor electronic data capture
CCMO: Central Committee on Research Involving Human Subjects
COVID-19: Coronavirus disease 2019
CPET: Cardiopulmonary exercise test
DICA: Dutch Institute for Clinical Auditing
DLCAs: Dutch Lung Cancer Audit — Surgery
eCRF: Electronic case report form
eGFR: Estimated glomerular filtration rate
EORTC QLQ-C30: European Organisation for Research and Treatment of Cancer, quality of life of Cancer patient’s module
EORTC QLQ-LC13: European Organization for Research and Treatment of Cancer, quality of life of cancer patients lung cancer module
EQ-5D-5L: EuroQol group, 5-level general health-related quality-of-life questionnaire
ERAS: Enhanced recovery after surgery
ESTS: European Society of Thoracic Surgeons
FFM: Fat-free mass
GDPR: General Data Protection Regulation
HADS: Hospital Anxiety and Depression Scale
HIIT: High-intensity interval training
iMCQ: IMTA Medical Consumption Questionnaire
IMT: Inspiratory muscle training
iMTA: Institute for Medical Technology Assessment
iPCQ: IMTA Productivity Cost Questionnaire
ISI: Insomnia Severity Index
ISO: International Organization for Standardization
MD: Medical doctor
MIP: Maximal inspiratory pressure
MMC: Máxima MC
MSc: Master of Science
MSEC: Maximum short exercise capacity
NSCLC: Non-small cell lung cancer
PG-SGA: Patient-Generated Subjective Global Assessment
PhD: Doctor of philosophy
SPIRIT: Standard Protocol Items: Recommendations for Interventional Trials
SRT: Steep ramp test
VO2max: Maximal oxygen uptake
